# Alfalfa Responses to Intensive Soil Compaction: Effects on Plant and Root Growth, Phytohormones and Internal Gene Expression

**DOI:** 10.3390/plants13070953

**Published:** 2024-03-26

**Authors:** Mingke Yan, Dongming Yang, Yijun He, Yonglong Ma, Xin Zhang, Quanzhen Wang, Jinghui Gao

**Affiliations:** 1 College of Grassland Agriculture, Northwest A&F University, Yangling 712100, China; 2 University of Chinese Academy of Sciences, Beijing 100049, China; 3 School of Agronomy, Ningxia University, Yinchuan 750021, China; 4 College of Plant Protection, Northwest A&F University, Yangling 712100, China

**Keywords:** soil compaction, *Medicago sativa* L., root responses, transcriptome, cell wall

## Abstract

The perennial legume alfalfa (*Medicago sativa* L.) is of high value in providing cheap and high-nutritive forages. Due to a lack of tillage during the production period, the soil in which alfalfa grows prunes to become compacted through highly mechanized agriculture. Compaction deteriorates the soil’s structure and fertility, leading to compromised alfalfa development and productivity. However, the way alfalfa responses to different levels of soil compaction and the underlying molecular mechanism are still unclear. In this study, we systematically evaluated the effects of gradient compacted soil on the growth of different cultivars of alfalfa, especially the root system architecture, phytohormones and internal gene expression profile alterations. The results showed that alfalfa growth was facilitated by moderate soil compaction, but drastically inhibited when compaction was intensified. The inhibition effect was universal across different cultivars, but with different severity. Transcriptomic and physiological studies revealed that the expression of a set of genes regulating the biosynthesis of lignin and flavonoids was significantly repressed in compaction treated alfalfa roots, and this might have resulted in a modified secondary cell wall and xylem vessel formation. Phytohormones, like ABA, are supposed to play pivotal roles in the regulation of the overall responses. These findings provide directions for the improvement of field soil management in alfalfa production and the molecular breeding of alfalfa germplasm with better soil compaction resilience.

## 1. Introduction

Alfalfa (*Medicago sativa* L.) is a high-quality forage with the largest planting area in the world. During the modern mechanized production of alfalfa, heavy loads of machinery and improper irrigation on the grassland can cause soil compaction. Moreover, throughout the entire production period, for a continuous 6–8 years after one planting with no rotation, the compaction state would continuously be intensified and impede the growth and development of alfalfa [1,2].

Defined as the increment in soil bulk density, soil compaction directly increases soil mechanical impedance, thus constraining plant root system penetration and development. Consequently, the reduced length, number and surface area of roots lead to impaired access to water, nutrients and air for the plant, as well as the interaction with various microbes [2,3,4]. Spreading to aboveground, it usually leads to compromised shoot growth, plant height and total biomass [2,5]. However, the compacted soil is not definitely a negative factor because it may turn out to be a growth facilitator in particular scenarios like low soil moisture [6], and in specific species such as narrow-leafed lupine and spring oilseed rape [7].

The root is the predominant organ for plant acclimation to soil-related stresses, including compaction. When encountering a hard clod, roots can employ either an obstacle-overcoming strategy, which is carried out by exerting greater axial and radial growth pressures to push or break the soil obstacles, or an obstacle avoidance strategy in which root growth is redirected to circumnavigate impenetrable obstacles [8]. Morphologically, it often manifests as an increased root diameter, root angles that are less steep and deflected root growth [3]. The more detailed physiological and biochemical reactions and molecular mechanisms underlying this process, in which phytohormone-regulated root cell development was proven to play pivotal roles, only began to be decoded recently. Facing mechanical impedance, reactive oxygen signaling integrated with ethylene and auxin responses is induced very early to mediate root growth changes in Arabidopsis, and the inhibition of ethylene responses allows improved root growth in response to barriers [9]. Touch-induced alterations in Ca^2+^, reactive oxygen species and pH signaling pathways are supposed to directly impact cell wall rigidity, therefore leading to root tropic responses to mechanical forces [10,11,12]. In rice, ethylene accumulates around the root tips in compacted soil, and it acts as a signal molecule to promote OsEIL1 (ETHYLENE INSENSITIVE 3-LIKE 1) activation, which directly up-regulates auxin biosynthesis through OsbZIP46 (basic REGION-LEUCINE ZIPPER 46) and OsYUC8 (YUCCA8), and indirectly promotes ABA biosynthesis in vascular tissues. The enhanced auxin and ABA responses further inhibit epidermal cell and root elongation in compacted soil conditions [13,14,15]. In Arabidopsis, AtFER (FERONIA) phosphorylates and stabilizes AtPIF3 (PHYTOCHROME-INTERACTING FACTOR 3), which regulates the expression of genes associated with the sloughing of root cap cells and root cap-specific genes including AtPIEZO, facilitating root penetration into the compacted soil [16,17]. Ethylene was supposed to orchestrate the AtFER-AtPIF3-AtPIEZO circuitry [17]. An integrated transcriptome and metabolome analysis of peanut root subjected to soil compaction revealed that differentially expressed genes (DEGs) and differentially accumulated metabolites related to soil compaction were mainly enriched in “oxidoreductase activity”, “lipid metabolism” and “isoflavonoid biosynthesis” pathways [18]. Schneider et al. [19] identified a root anatomical characteristic in maize, wheat and barley named multiseriate cortical sclerenchyma, which was formed upon exogenous ethylene exposure and associated with improved root tensile strength and increased penetration ability in compacted soils.

Due to the perennial nature of alfalfa, the grassland soil strength tends to increase year after year under farm machinery traffic and water permeation during field operations. Previous studies revealed that soil compaction resulted in a significant shoot biomass reduction in alfalfa in the first year [1,20,21,22]. If the heavy load is only applied once and not any more in the following years, the soil will loosen, and the forage yield will be restored, indicating the potential employment of alfalfa as a candidate crop to improve compacted soil [20,21,23]. These studies revealed the high value of identifying the key characteristics of the alfalfa root system architecture and underlying molecular mechanism countering soil compaction stress. However, research to date has only focused on descriptions of a few cultivation responses of the alfalfa plant in the field; therefore, deep insights are urgently needed for efficient genetic study and breeding works.

In this study, we analyzed in detail the effects of different levels of soil compaction on alfalfa growth and further confirmed the effects in more cultivars. We especially focused on the root system structure alterations. Transcriptome, anatomy and physiological approaches were further employed to elucidate the underlying stress-responsive mechanisms. We found that intensified soil compaction displayed a significant adverse effect on alfalfa growth, and a set of genes related to cell wall development and phytohormone regulation was illustrated in the processes. To the best of our knowledge, this work is the first attempt at linking the morphologic, anatomic and molecular responses of alfalfa subjected to soil compaction.

## 2. Materials and Methods

### 2.1. Soil Preparation

The soil used in this study was farmland loessal soil collected at Yangling, China (34°18′08″ N, 108°04′56″ E). Physicochemical properties of the soil were analyzed according to NY/T 1121 standard series (National Agriculture Industry Standard of China) and are listed in Table S1. The soil was sieved at 4 mm and placed in round pots with a 17 cm diameter and 11.5 cm height. Four levels of compaction treatments were reached by manually controlling soil bulk density by pressing soil with escalating dry weight with the same volume. The control pots were left uncompacted (1.2 g cm^−3^, CK), and gradient compaction treatments were set as CP1.3 (1.3 g cm^−3^), CP1.4 (1.4 g cm^−3^) and CP1.5 (1.5 g cm^−3^, also referred to as CP excluding the gradient analysis, which is close to the upper limit caused by field machinery [24]). Three biological replicates (at least three pots for each with 15–20 seedlings in one pot, cultivated in different growth chambers) were performed for each treatment. Soil hardness (listed in Table S1) was measured with a soil penetrometer (TYD-2; Zhejiang TOP Instrument Co., Hangzhou, China), with the tip cone and the driving shaft vertically inserted into the soil (soil moisture content is 26.2%) for 7–8 cm at a constant rate of about 30 mm s^−1^.

### 2.2. Plant Growth

For seeds of alfalfa cultivars used in this study, “Bara420YQ”, “WL363HQ” and “WL366HQ”, were obtained from Beijing Rytway Seed Co., Ltd. (Beijing, China), and “Zhongmu-1”, “Zhongmu-4” were obtained from Beijing Best Grass Industry Co., Ltd. (Beijing, China). Healthy seeds were sown in the pots with soil treatments described above and then covered with about 0.5 cm loosened soil to promote simultaneous germination by keeping a moist environment. Seed germination and plant growth were carried out in growth chambers with a 16 h light/8 h dark photoperiod and a temperature of 25/20 °C (light/dark). Photosynthetic photon flux density of the light was 300 µmol m^−2^ s^−1^. The soil was carefully watered with tap water to keep the field moisture capacity close to 70%. After six (study for gradient levels of compaction effect) or five weeks (other analysis), shoots and roots of the plants were harvested separately for corresponding analysis.

### 2.3. Sampling and Morphological and Physiological Analysis

For analysis of root system structure, intact roots were separated and cleaned from soil by soaking and washing using tap water. The fresh roots were then manually unraveled and scanned using a ScanMaker i850 scanner (Microtek, Shanghai, China). The obtained images were loaded into RhizoVision Explorer [25] for analysis of root number, length, diameter, volume, surface area and root orientation. Three biological replicates, with each one imaging five independent roots, were performed for each treatment. For determination of dry weight and element contents, fresh roots were rinsed using deionized water, dried in an oven at 105 °C for 20 min immediately after sampling and then kept at 65 °C at a constant weight. Three biological replicates were performed for each treatment.

Determination of nitrogen, phosphorus and potassium contents was performed as previously described [26]. Briefly, the dry samples were pulverized, and about 0.15 g of powder was thoroughly digested via H_2_SO_4_-H_2_O_2_ method. Part of the obtained solutions was loaded onto a flow injection autoanalyzer (FlowSys, Systea, Rome, Italy) to colorimetrically determine the concentrations of nitrogen and phosphorus, and the rest of the solutions was loaded onto an atomic absorption spectrometer (Perkin Elmer-PinAAcle 900F, Waltham, MA, USA) to measure potassium concentration by flame emission spectrometry.

For RNA and phytohormone extraction, middle and lower sections of roots (3–4 cm below soil surface), which ruled out possible surface soil loosening, were manually collected from the soil and quickly washed using deionized water. After quickly absorbing remaining water, the roots were immediately frozen in liquid nitrogen and stored at −80 °C until extraction process. Because non-uniformity might occur during sampling owing to the quick separation of fragmented root sections, five biological replicates for RNA-Seq and at least four replicates for phytohormone analysis were performed for each treatment to ensure data reliability.

### 2.4. RNA Extraction, Transcriptome Sequencing and Quantitative Real-Time PCR (qRT-PCR) Analysis

Total RNA was extracted using RNAprep Pure Plant Kit (Tiangen Biotech, Beijing, China) according to the manufacturer’s instructions. After concentration and quality examination, mRNA was purified from total RNA using poly-T-oligo-attached magnetic beads for transcriptome sequencing. The following fragmentation, sequencing library construction and qualification processes were performed using NEBNext Ultra RNA Library Prep Kit for Illumina (NEB, Ipswich, MA, USA) according to the manufacturer’s instructions. The qualified libraries were pooled and sequenced according to effective library concentration with PE150 strategy on Illumina NovaSeq 6000 platform in Novogene Bioinformatics Technology Co., Ltd. (Beijing, China).

For qRT-PCR analysis, qualified total RNA was reverse-transcribed into cDNA using a HiScript II 1st Strand cDNA Synthesis Kit (+gDNA wiper) (Vazyme Biotech Co., Ltd., Nanjing, China). qRT-PCR amplifications were conducted using PerfectStart^®^ Green qPCR SuperMix (TransGen Biotech, Beijing, China) on a LightCycler480 II thermal cycler (Roche, Rotkreuz, Switzerland). DEGs were randomly selected from the key processes of lignin and flavonoid biosynthesis network, and from auxin and ABA transduction networks, as described below, with the *β-ACTIN* gene being used as an internal reference [27]. Four replicates were performed for each treatment. The primers used in this study are listed in Table S2.

### 2.5. Bioinformatics Analysis

The original fluorescence image files generated from sequencer were transformed to short reads (raw data) by base calling and recorded in FASTQ format. Quality control, adapter trimming and quality filtering of the raw sequences were processed via Fastp (version 0.23.1) [28] to obtain clean reads. The clean reads from each sample were separately aligned to the newly published reference genome of alfalfa cultivar, Zhongmu-4 [29], using hisat2 (version 2.2.1) [30] with the default settings for paired-end reads. The output SAM files from alignment were then sorted and transformed to BAM format via samtools (version 1.16.1). Next, FeatureCounts (integrated in subread package, version 2.0.3) was employed to count the reads mapped to each gene, with “-M --fraction” options being specified to make a fractional count of the multi-mapping fragments and considering the high duplicate attribute of the alfalfa genome as an autotetraploid.

The R package DESeq2 (version 1.40.2) was used to normalize the read counts over different samples and evaluate the gene expression difference between treatments [31]. A gene was considered to be significantly differentially expressed when the normalized read counts changed by more than twofold (|log2(fold change)| > 1) and the adjusted *p*-value was less than 0.05. Subsequently, Gene Ontology (GO) and Kyoto Encyclopedia of Genes and Genomes (KEGG) pathway annotation of the genes were assigned using a web tool eggNOG-mapper (version 2.1.12), with the taxonomic scope being restricted in “Viridiplantae—33090” [32]. The assigned annotations were then enriched using R package clusterProfiler (version 4.8.3), with all the expression-detected genes in this study set as the background [33]. To reduce the redundancy of GO enrichment results, the significantly enriched GO annotations (number of enriched genes > 3 and adjusted *p*-value < 0.05) were further clustered using GOMCL (version 0.0.1) and visualized via Cytoscape [34].

### 2.6. Root Sectioning and Microscopy

Root anatomical slides were obtained by cross-sectioning the roots using the paraffin embedding method with safranin and fast green staining. Briefly, one-centimeter root sections from mature roots were fixed in freshly prepared formalin solution (5% formalin, 5% acetic acid and 45% ethenol) for 24 h. Fixed root sections were washed with 1 × PBS buffer three times and kept in ClearSee solution. After dehydrating in a graduated ethanol–xylenes solution series, the fixed samples were infiltrated with and embedded in paraffin. Paraffin sections with a thickness of 10 μm were cut using the RM2016 microtome fitted with Leica 819 disposable blades (Leica, Wetzlar, Germany). The sections were subsequently floated on 42 °C water bath for relaxing compression and mounting on the B004 microscope slide (Ebiogo, Hefei, China). Afterwards, dried sections were de-paraffinized, re-hydrated through a graded xylene-ethanol-water series, stained with Safranin O and Fast green solution (Ebiogo, Hefei, China) and vitrified by dimethylbenzene. The obtained slides were finally sealed with neutral gum and imaged with a BX53 microscope (Olympus, Tokyo, Japan).

ImageJ was used to measure the area (section, xylem and vessel) and cell number in the section images. Xylem area percentage was calculated as the area percentage of xylem per cross section. Vessel area percentage was calculated as the area percentage of total vessels per xylem region per cross section, and vessel density was calculated as the number of total vessels in the whole xylem area per cross section.

### 2.7. Extraction and Assay of Phytohormones

For extraction of phytohormones, each root sample was ground into homogenate in nitrogen bath, and 50–75 mg of powder was accurately weighed and transferred to a 2 mL test tube. Immediately, 1 mL of ethyl acetate was added into the tube to extract hormones. After shaking at 2000 rpm for 10 min and centrifuging at 12,000× *g* for 10 min, the supernatant was transferred to a new tube and dried in a centrifugal concentrator. To dissolve the hormones, 175 μL of 50% methanol was added into the tube followed by shaking at 2000 rpm for 10 min and centrifuging at 13,000× *g* for 15 min. Finally, the supernatant hormone solution was filtered by 0.22 μm hydrophobic filter membranes and loaded onto a liquid chromatography–quadrupole ion trap–mass spectrometry (LC–QTRAP–MS) (QTRAP 5500, AB SCIEX, Foster City, CA, USA) system for phytohormone concentration determination through comparison with corresponding standard solutions.

### 2.8. Statistical Analysis

F-test, one-way ANOVA test, and Student’s *t*-test in the R package stats were employed to analyze the differences between multi- or two treatments. Coupled with an ANOVA test for multi-treatments, significant differences were identified at the 0.05 probability level by least significant difference (LSD) test provided in the agricolae package (version 1.3-6) in R. All results were presented as the mean ± SE from at least 3 independent biological replicates.

## 3. Results

### 3.1. Effects of Different Levels of Soil Compaction on Alfalfa Growth

**Figure 1:**
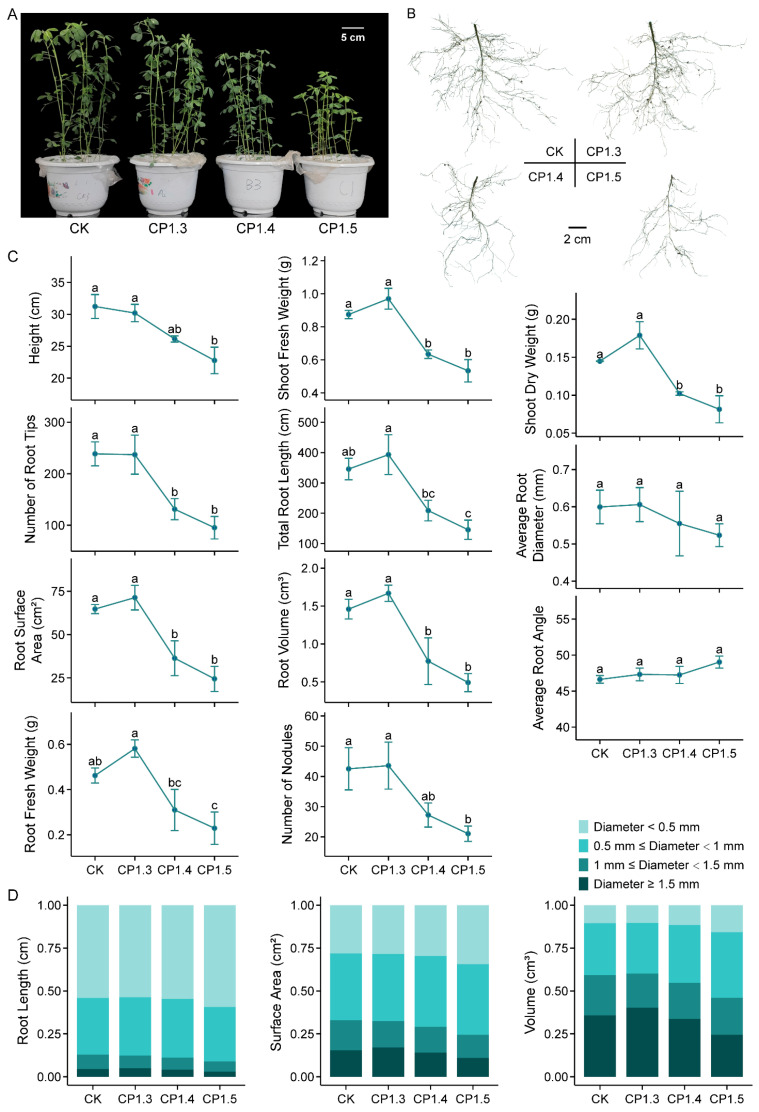
Effects of different levels of soil compaction on alfalfa seedling growth. (**A**) Shoot phenotype. (**B**) Root image obtained by root scanner. (**C**) Growth parameters of seedlings: height, shoot fresh weight, shoot dry weight, number of root tips, total root length, average root diameter, root surface area, root volume, average root angle, root fresh weight and number of nodules. (**D**) Distribution of root length, surface area and volume upon different root diameters. Seedlings of Zhongmu-1 were grown in soils with different bulk densities for six weeks until imaging and measurement; *n* = 3 sets of at least 5 seedlings. CK, CP1.3, CP1.4 and CP1.5 correspond to soil bulk densities of 1.2 g cm^−3^, 1.3 g cm^−3^, 1.4 g cm^−3^ and 1.5 g cm^−3^, respectively. Error bars represent the means ± SE. Different letters represent significant differences (*p* < 0.05) analyzed using the least significant difference test.

In order to evaluate the effects of different levels of soil hardness on alfalfa growth, we checked the seedling behavior upon four compaction gradients marked by the soil bulk density of the Zhongmu-1 cultivar. As indicated in Figure 1A–C, when other environmental factors were suitable for alfalfa growth, a slight compaction (CP1.3) of the soil displayed a positive effect compared with CK (non-compacted). When the compactness intensified, a growth reduction appeared in both the shoot and root. When the soil bulk density reached 1.5 g cm^−3^, the shoot height was reduced by 27.0%, the biomass was reduced by 43.7%, and the number, length, diameter, surface area and volume of roots were reduced by 60.0%, 57.9%, 12.7%, 62.2% and 66.3%, respectively. Correspondingly, less mature nodules were developed. The average root angle tended to increase when the soil was compacted, although the present data were not significant. More specifically, the severe compaction of soil not only resulted in reductions in the total root length, surface area and volume, but also rearranged the percentage distribution of thin and thick roots (Figure 1D). For example, the percentages of root length, surface area and volume for a root with a diameter of less than 0.5 mm increased from 54.2%, 28.2% and 10.5% in CK to 59.3%, 34.4% and 15.7% in CP1.5, while those for a root with a diameter of more than 1.5 mm decreased from 4.5%, 15.4% and 35.8% in CK to 3.0%, 10.9% and 24.5% in CP1.5, respectively.

**Figure 2:**
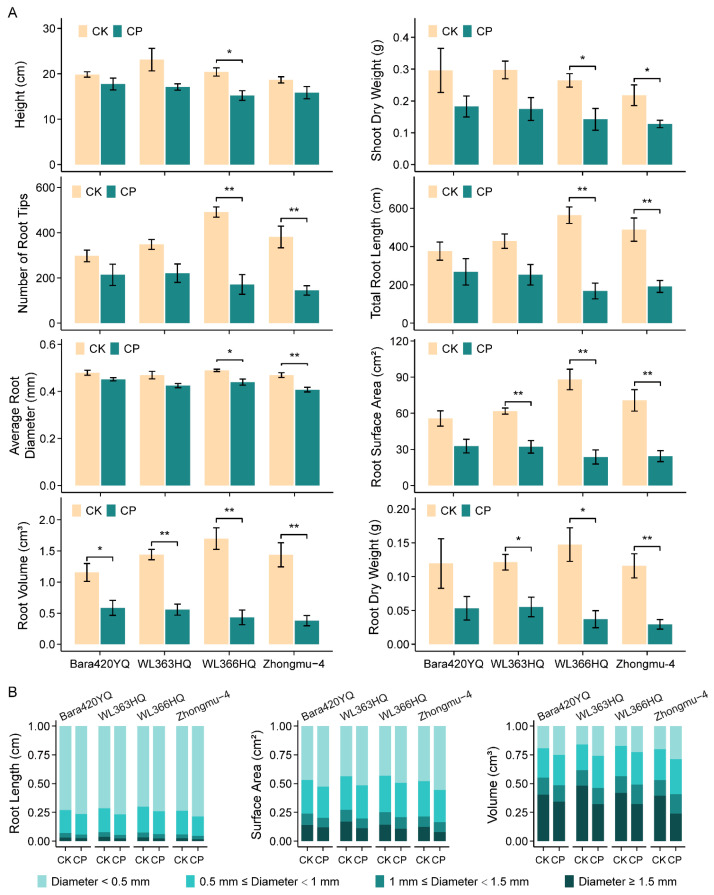
Behaviors of different cultivars of alfalfa seedling under soil compaction stress. (**A**) Growth parameters: height, shoot dry weight, number of root tips, total root length, average root diameter, root surface area, root volume and root dry weight. (**B**) Distribution of root length, surface area and volume upon different root diameters. Seedlings of different cultivars were grown in soil with bulk densities of 1.2 g cm^−3^ (CK) and 1.5 g cm^−3^ (CP) for five weeks until imaging and measurement; *n* = 3 sets of at least 5 seedlings. Error bars represent the means ± SE. Asterisks represent significant differences between samples analyzed using Student’s *t*-test: * *p* < 0.05; ** *p* < 0.01.

The negative effect of soil compaction was further confirmed over four additional cultivars, namely Bara420YQ, WL363HQ, WL366HQ and Zhongmu-4. As demonstrated in Figure 2, all seedling growth was hampered in highly hardened soil when compared with the control. Moreover, comparisons between cultivars revealed different seedling performances. Overall, the Bara420YQ and WL363HQ seedlings behave better than WL366HQ and Zhongmu-4 in compacted soil, as indicated by both shoot and root growth. This trend was specifically contrasted in the number, length and diameter of roots and the resulting root surface area and volume (Figure 2A and Figure S1). The shrinkage of root development was mainly a result of thick root (diameter ≥ 1.5 mm) reduction and thin root (diameter < 0.5 mm) expansion (Figure 2B).

### 3.2. Transcriptome Profile of Alfalfa Root Response to Soil Compaction

**Figure 3:**
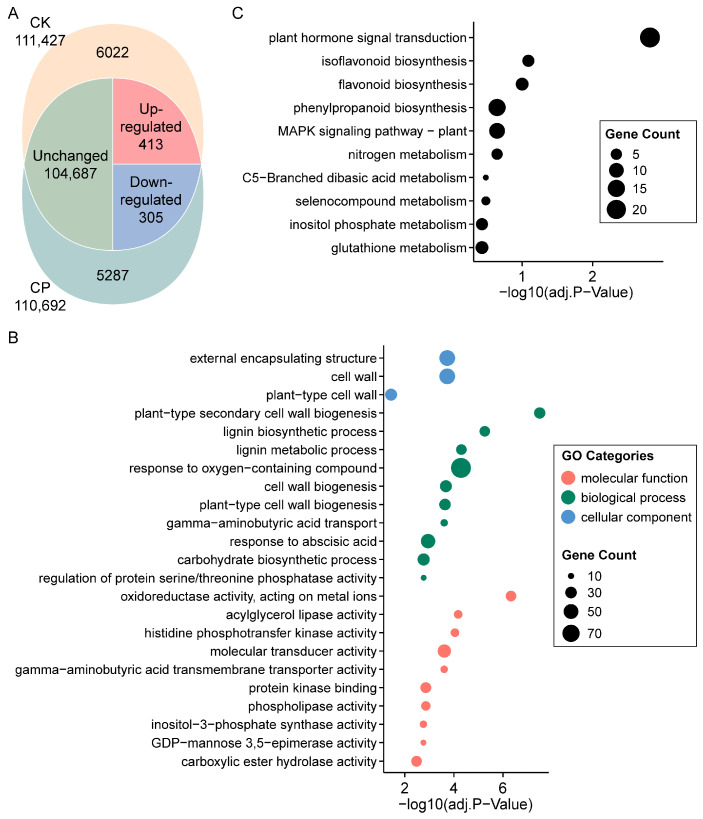
Expression summary and function enrichment analysis of genes from alfalfa root in response to soil compaction. (**A**) Venn diagram of overlap of total and differentially expressed genes detected in each sample. (**B**) Gene Ontology enrichment results of differentially expressed genes (top 10 significantly enriched annotations are displayed). (**C**) KEGG pathway enrichment results of differentially expressed genes. CK, 1.2 g cm^−3^ (bulk density); CP, 1.5 g cm^−3^.

To obtain a comprehensive understanding of the gene expression alterations in alfalfa root responses to soil compaction, the control and soil-compaction-stressed root samples of Zhongmu-4 were submitted to an RNA-Seq analysis. The Zhongmu-4 cultivar was selected because its reference genome was the newest and finest-assembled for alfalfa to date [29]. As indicated in Figure 3A, total RNA sequencing detected 111,427 and 110,692 expressed genes in the control and CP, in which 6022 and 5287 genes were exclusively expressed, respectively. For genes expressed in both conditions, 413/305 were significantly up-/down-regulated, and 104,687 stayed unchanged (or not significantly changed) when comparing CP to CK (Table S2).

To explore the structural alterations and undergoing metabolic mechanisms of alfalfa root subjected to severe soil compaction, the DEGs, including genes specifically expressed or co-expressed but significantly up-/down-regulated in CK and CP, were assigned with GO and KEGG pathway annotations, and the annotations were further enriched by the relative gene number in each entry (Figure 3B,C and Table S3). The GO annotation revealed that the gene products were pronouncedly localized at the external encapsulating structure, mainly the cell wall, which was further supported by the most significantly enriched biological process, namely “plant-type secondary cell wall biogenesis”, and the closely related “lignin biosynthetic process”. Other enriched specialized processes included the “response to oxygen-containing compound”, “gamma-aminobutyric acid transport”, “response to abscisic acid”, “carbohydrate biosynthetic process” and “regulation of protein serine/threonine phosphatase activity”. In addition, the predominant outlined molecular function of the DEGs were “oxidoreductase activity, acting on metal ions”, “acylglycerol lipase activity” and “histidine phosphotransfer kinase activity”. For the KEGG pathway, “plant hormone signal transduction”, “isoflavonoid biosynthesis” and “flavonoid biosynthesis” were the most significantly enriched.

**Figure 4:**
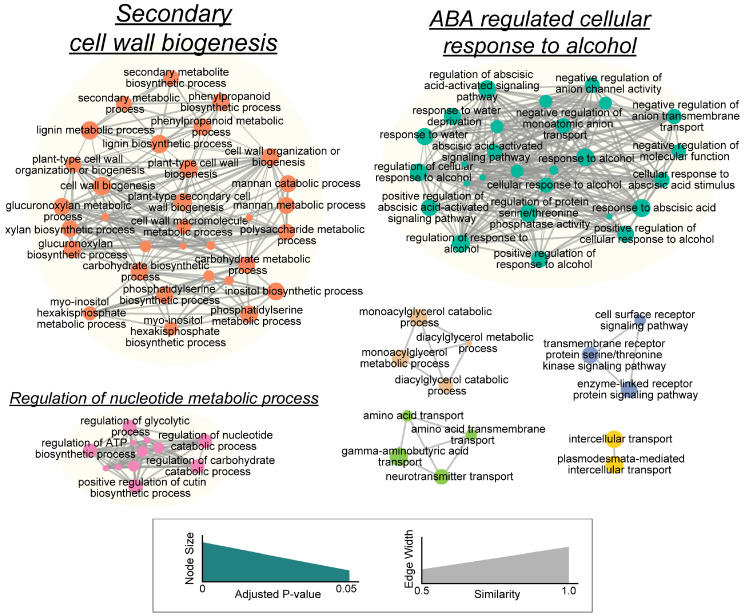
The lustering of the significantly enriched Gene Ontology biological process annotations. The nodes represent gene sets within each GO term, and the width of the line (grey edge) is proportional to the number of overlapping genes between the two nodes. The clusters of functionally related gene sets are circled, and the labels (underlined) are manually assigned based on the word frequency. The detailed cluster component is also described in Table S6. The size of the cluster label is relative to the number of nodes in the cluster.

Because the primitive lists of overrepresented GO terms were too large and contained redundant overlapping GO terms that hindered the informative functional interpretations, non-redundant associations were extracted based on the overlap of gene members between GO biological process terms. As shown in Figure 4, the DEGs mainly functioned in three clusters, namely “secondary cell wall biogenesis”, “ABA-regulated cellular response to alcohol” and the “regulation of nucleotide metabolic process”.

The combination of annotations of the GO and KEGG pathways indicates that the cell wall architecture, flavonoids and lignin biosynthesis might have been modified, and phytohormones like ABA might play pivotal roles in the alfalfa responses to soil compaction. To validate the RNA-Seq data, the relative expression levels of eight genes with specific primers were checked via qRT-PCR. Except for one gene–primer- pair with a very low amplification efficiency, the expression patterns of all other seven genes displayed high concordance with the RNA-Seq results (Figure S3). Therefore, the detailed gene expression and the corresponding anatomic or physiological variation in these processes were further probed afterwards.

### 3.3. Adapted Phenylpropanoid-Derived Pathways and Root Anatomy under Soil Compaction

**Figure 5:**
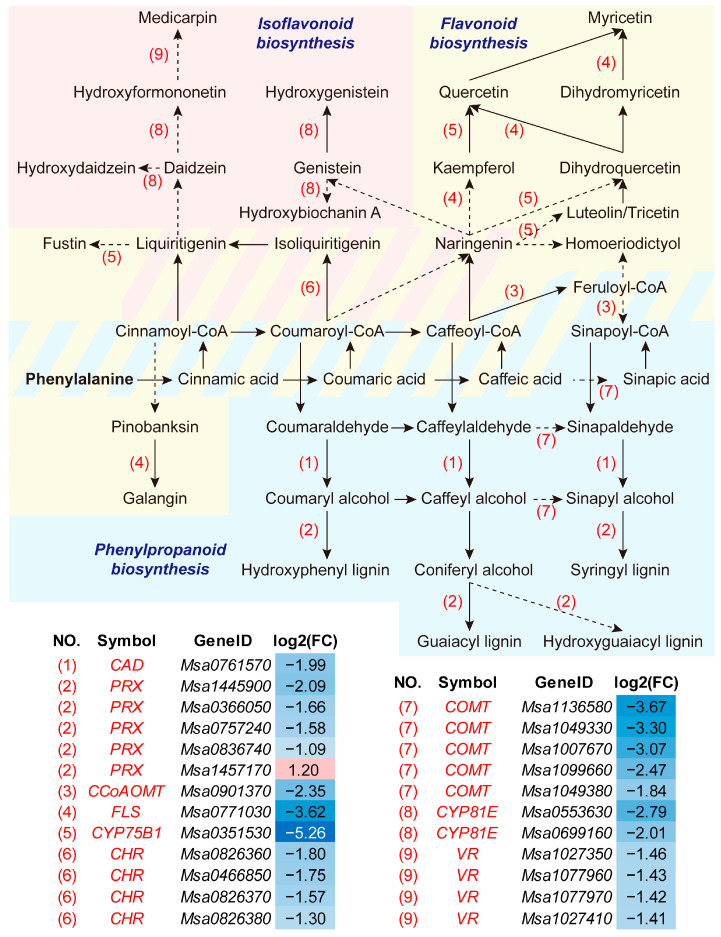
Core genes differentially expressed in phenylpropanoid, flavonoid and isoflavonoid biosynthesis pathways. Solid arrows represent direct reactions, and dashed arrows represent indirect reactions. CAD, CINNAMYL ALCOHOL DEHYDROGENASE; CCoAOMT, CAFFEOYL CoA 3-O-METHYLTRANSFERASE; CHR, CHALCONE REDUCTASE; COMT, CAFFEATE/5-HYDROXYFERULATE 3-O-METHYLTRANSFERASE; CYP75B1, flavonoid 3′-monooxygenase; CYP81E, isoflavone/4′-methoxyisoflavone 2′-hydroxylase; FC, fold change (CP/CK) of expression level; FLS, FLAVONOL SYNTHASE; PRX, PEROXIDASE; VR, VESTITONE REDUCTASE.

The biosynthetic pathways of flavonoid, isoflavonoid and lignin were interconnected. As illustrated in Figure 5, nearly all DEGs in the combined network were down-regulated when the alfalfa root suffered soil compaction. The key gene homologs included *CAD* (*CINNAMYL ALCOHOL DEHYDROGENASE*), *COMT* (*CAFFEATE/5-HYDROXYFERULATE 3-O-METHYLTRANSFERASE*), *CCoAOMT* (*CAFFEOYL COA 3-O-METHYLTRANSFERASE*) and *PRX* (*PEROXIDASE*) in phenylpropanoid and lignin biosynthesis; *FLS* (*FLAVONOL SYNTHASE*), *CYP75B1* (*FLAVONOID 3′-MONOOXYGENASE*) and *CHR* (*CHALCONE REDUCTASE*) in flavonoid biosynthesis; and *CYP81E* (*ISOFLAVONE/4′-METHOXYISOFLAVONE 2′-HYDROXYLASE*) and *VR* (*VESTITONE REDUCTASE*) in isoflavonoid biosynthesis.

**Figure 6:**
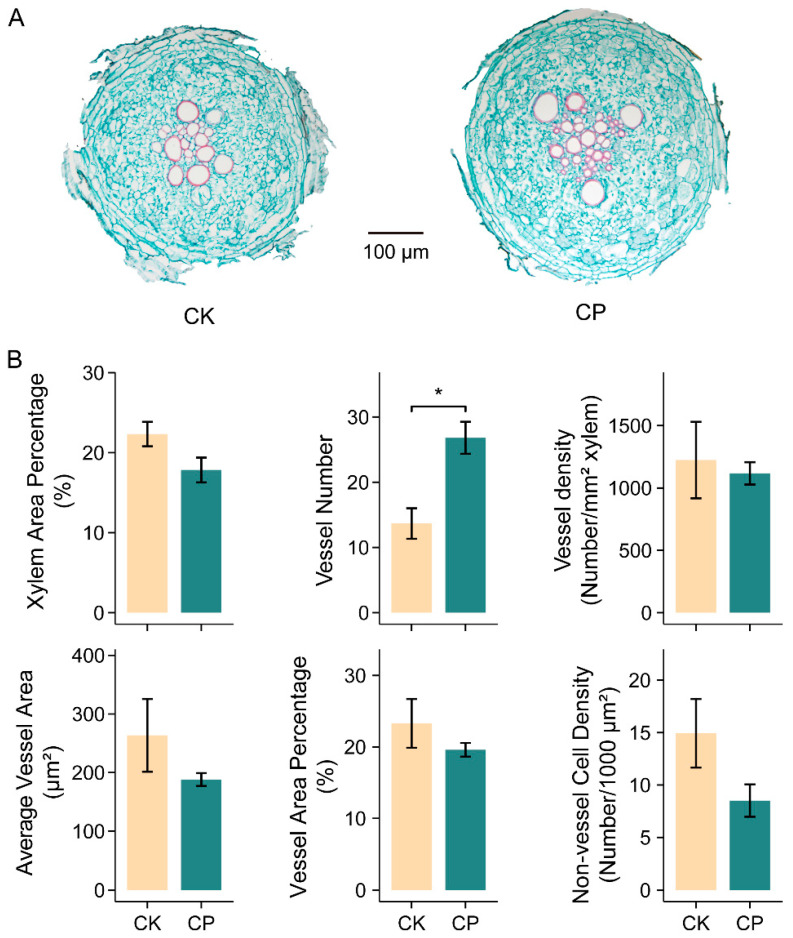
Anatomical analysis of alfalfa root responses to soil compaction. (**A**) Root cross sections. Slices were obtained via paraffin embedding method with safranin and fast green staining. (**B**) Statistics of root sections: xylem area percentage, vessel number, vessel density, average vessel area, vessel area percentage and non-vessel cell (live cell) density. Seedlings of Zhongmu-1 were grown in soil with bulk densities of 1.2 g cm^−3^ (CK) and 1.5 g cm^−3^ (CP) for five weeks until imaging; *n* = 3 sets of at least 3 roots. Error bars represent the means ± SE. Asterisks represent significant differences between samples analyzed using Student’s *t*-test: * *p* < 0.05.

Considering the large set of DEGs related with the cell wall, especially lignin biogenesis and secondary cell wall establishment, we conjectured that the cell structure might be modified when the alfalfa roots were faced with compacted soil. We thus checked the root anatomy variation in the seedlings grown under the CK and CP treatments. A noticeable change in the root anatomy was the doubled vessel number under the CP treatment, which also led to an increase in the total vessel area. Additionally, the xylem area percentage, average vessel area, vessel area percentage and the density of the living cells, including the phloem, pericycle, endodermis and cortex, were slightly decreased, although this was insignificant in the present study (Figure 6).

### 3.4. Alterations in Phytohormone Biosynthesis

**Figure 7:**
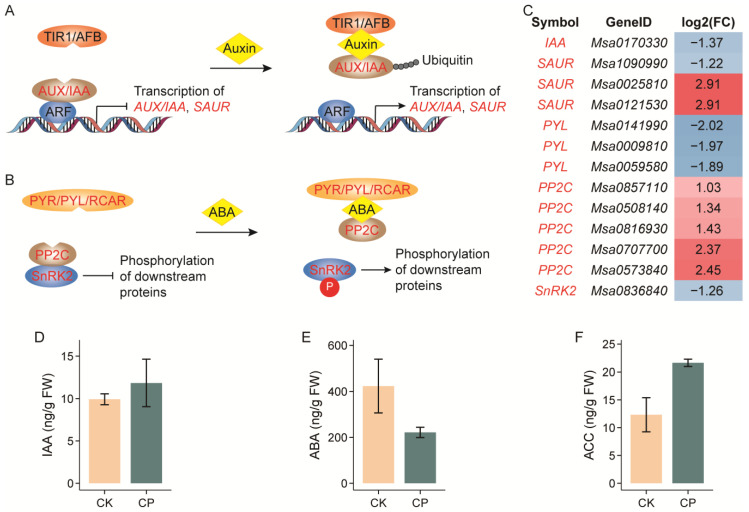
Biosynthesis and accumulation of key phytohormones related to soil compaction responses in alfalfa roots. (**A**–**C**) Core genes differentially expressed in auxin and ABA signaling pathways. (**D**–**F**) Contents of auxin, ABA and ethylene precursor 1-aminocyclopropane-1-carboxylic acid (ACC). ARF, AUXIN RESPONSE FACTOR; AUX, AUXIN; IAA, INDOLEACETIC ACID; FC, fold change (CP/CK) of expression level; PP2C, TYPE 2C PROTEIN PHOSPHATASE; PYL, PYRABACTIN RESISTANCE-LIKE; PYR, PYRABACTIN RESISTANCE; RCAR, REGULATORY COMPONENT OF ABA RECEPTORS; SAUR, SMALL AUXIN UP-REGULATED RNA; SnRK2, SUCROSE NON-FERMENTING 1 (SNF1)-RELATED PROTEIN KINASES 2; TIR1, TRANSPORT INHIBITOR RESPONSE 1; AFB, AUXIN SIGNALING F-BOX. Seedlings were grown in soil with bulk densities of 1.2 g cm^−3^ (CK) and 1.5 g cm^−3^ (CP) for five weeks until imaging; *n* = 5 sets of whole roots. Error bars represent means ± SE (no significant differences found in (**D**–**F**) at *p* < 0.05 via Student’s *t*-test).

The expression of a set of genes in phytohormone signaling pathways was also noticeably changed. For example, *AUX/IAA* (*AUXIN*/*INDOLEACETIC ACID*) and *SAUR* (*SMALL AUXIN UP-REGULATED RNAs*) function in auxin signaling, and the transcription of the *IAA* homologous gene was repressed by more than 2.5 times, while different *SAUR* homologs were diversely regulated. PYR/PYL/RCAR (PYRABACTIN RESISTANCE/PYRABACTIN RESISTANCE-LIKE/REGULATORY COMPONENT OF ABA RECEPTORS), PP2C (TYPE 2C PROTEIN PHOSPHATASE) and SnRK2 (SUCROSE NON-FERMENTING 1 (SNF1)-RELATED PROTEIN KINASES 2) are critical components in the ABA signal cascade, in which the expressions of the *PYL* and *SnRK2* gene homologs were repressed by nearly 4.0 and 2.4 times, while the *PP2C* homologs were generally induced under the CP treatment (Figure 7A–C).

While taking into account the key roles played by phytohormones during plant adaptation to soil compaction, we further examined the contents of auxin, ABA and ethylene, as well as cytokinin, gibberellin, salicylic acid (SA) and jasmonic acid (JA) in alfalfa roots subjected to soil compaction (Figure 7D–F and Figure S4). Unfortunately, although five repeats were performed, we only found a significant elevation in the JA content in the stressed roots (1.84 times). Nevertheless, there was a moderate decrease in the ABA content and increases in the ethylene precursor 1-aminocyclopropane-1-carboxylic acid (ACC) and trans-Zeatin-riboside contents.

## 4. Discussion

### 4.1. Effects of Soil Compaction on Alfalfa Growth Depend on Stress Severity and Plant Germplasm

Soil compaction is generally thought to be an adverse factor that restrains plant growth except in some specific environmental conditions, such as low soil moisture [6], or a few species such as narrow-leafed lupine and spring oilseed rape [7]. In this study, we found that the effects of soil compaction and alfalfa growth did not simply have a monotonic relationship, but depended on the compaction intensity. Moderate compaction (1.3 g cm^−3^ in the present study) was beneficial for the growth of both the shoots and roots of alfalfa. This might be due to facilitated root contact with soil particles, which improved the plant’s access to water and nutrition, and also root anchorage. However, if the soil was severely compacted (over 1.4 g cm^−3^ in the present study), the overall negative effects would overwhelm its benefits on alfalfa (Figure 1). The compromised development of the shoots and roots was in accordance with most species, such as barley, wheat, oat, peas, sugar beet and horse bean [35]. Nevertheless, the average root diameter was more or less decreased in intensively compacted soil in all cultivars, which is inconsistent with several other crops including Brazilian pine, *Quercus pyrenaica* and *Fraxinus angustifolia* [36,37,38,39]. The dramatically increased soil bulk density and strength and reduced soil porosity and hydraulic properties were supposed to be the culprits [2]. These results suggest that it is of great value to keep the soil bulk density less than 1.3 g cm^−3^ to cultivate alfalfa in soils with similar properties as those in the present study.

High-level soil compaction displayed universal repression on different alfalfa cultivars. However, there is a prospect to ameliorate the damage by improving the germplasm besides field management because the repression effects were different across cultivars (Figure 2 and Figure S1). Strategies to improve the soil compaction tolerance germplasm could be derived from other species [7] or from cultivars with better performance like in the current study. Furthermore, the large-scale screening and selection of superior germplasms and digging into the variety characteristics in the future are the recommended directions.

### 4.2. Phenylpropanoid, Lignin, Flavonoid and Cell Wall Modification in Alfalfa Root Responses to Soil Compaction

The phenylpropanoid metabolism pathway links the central carbon metabolism (primary metabolism) to specialized metabolism and gives rise to crucial metabolites, including lignin and flavonoids. Lignin was mostly deposited in the secondary cell walls of specialized cell types, and its predominant function is enhancing the mechanical strength and rigidity of the cell wall and facilitating xylem vessel formation to transport water and nutrients over a long distance [40]. The structures of newly synthesized lignin polymers seem to largely depend on the type and intensity of stress and on the plant species, and the control of stress-related lignification also involves several regulatory levels including transcription [41]. The present study displayed a synergetic expression decline in genes involved in lignin biosynthesis (*CAD*, *COMT*, *CCoAOMT* and *PRX*) in CP-treated roots, which was supposed to reduce lignin deposit in the cell wall and xylem vessels (Figure 5). The reduction in the lignin content was reported to result in impaired secondary cell wall integrity and reduced stem height and biomass, leading to growth inhibition in alfalfa and other plants [42,43,44]. The root sections in the present study showed that the vessel number was significantly increased in the roots grown in compacted soil, but the average vessel area, total vessel area and xylem area percentages were slightly decreased (Figure 6), indicating that alfalfa roots tend to develop more thinner vessels in response to compaction stress. This is overall in line with the changes discovered in *Fraxinus angustifolia* [39]. The xylem vessel is principally made up of lignin. The vessel diameter determines the xylem’s area-specific conductivity, and thicker root vessels implicate higher hydraulic conductivity, which is a key indicator of the roots’ ability to transport water from soils [45,46]. Therefore, the soil-compaction-stressed alfalfa roots likely suffered from impaired water transport capacity. Apart from xylem vessels, the altered cell development might partly be caused by inhibited lignification and, in turn, hindered root and plant growth, as anatomical xylem vessel development was proposed to correlate with the plant height and growth rate in herbaceous plants [47].

Flavonoids (including isoflavonoids) are an important class of plant secondary metabolites, and some of them play vital roles in regulating plant development and responses to stresses such as drought, high temperatures, cold temperatures, salinity, radiation and heavy metals [48,49]. Flavonoids such as echinatin, liquiritigenin, methoxychalcone, formononetin and medicarpin were also crucial signaling molecules in alfalfa–rhizobia interactions, which could stimulate the nodulation of rhizobia [50,51]. In the current investigation, a set of genes involved in flavonoid and isoflavonoid biosynthesis were down-regulated simultaneously in the roots under the CP treatment (Figure 5). Similar results were also found in peanut roots subjected to compacted soil [18]. Whether these genes are inducements for the inhibition of root or nodule development or both needs further elucidation.

Transcription regulatory networks for the biosynthesis of flavonoids and lignin share certain specific regulators, such as transcription factors, mediators and microRNAs [52]. Consequently, modulating some of the network components may affect how other processes are carried out (Figure 5). For example, lignin modification via the down-regulated lignin biosynthesis pathway gene *HCT* (*HYDROXYCINNAMOYL-COA SHIKIMATE/QUINATE HYDROXYCINNAMOYL TRANSFERASE*) is associated with increased levels of gibberellins and certain flavonoid compounds in roots, and it leads to an increased nodule number in alfalfa by up to 3.5 times [53]. Therefore, future research devoted to thoroughly interpreting the function of soil-compaction-responsive core DEGs is necessary and complicated.

### 4.3. Plant Hormone Networks Involved in Alfalfa Root Responses to Soil Compaction

Phytohormones coordinately regulate plant growth and development, including roots’ acclimation to compacted soil. Among the hormones, auxin, ABA and ethylene are the most frequently quoted. When roots grow into compacted soil, ethylene orchestrates the induction of both auxin and ABA biosynthesis to regulate root elongation [13,14,15]. The current study revealed a noticeable variation in plant hormone contents between five replicates of plants, which resulted in a moderate decrease and increase in ABA and the ethylene precursor ACC contents, respectively (Figure 7D–F). For significant DEG enrichment in plant hormone signal transduction, the auxin and ABA signaling pathways swept the largest gene sets (Figure 4 and Figure 7A–C).

In the auxin signaling machinery, *AUX*/*IAA* and *SAURs* were differentially regulated. Both gene families are early and primary auxin-responsive genes. Auxin promotes the interaction between the TIR1/AFB (TRANSPORT INHIBITOR RESPONSE 1/AUXIN SIGNALING F-BOX) and AUX/IAA proteins, resulting in the degradation of AUX/IAAs and the release of *ARF* (*AUXIN RESPONSE FACTOR*) repression [54]. SAURs play a central role in auxin-induced acid growth but can also act independently of auxin. Several *SAUR* genes have been shown to play roles in diverse processes of plant growth, development and stress responses. For example, *AtSAUR19*, *AtSAUR63* and *AtSAUR41* promote cell expansion, and *AtSAUR41* is inducible by ABA to modulate salt tolerance in Arabidopsis [55,56,57]. Additionally, the expression of an auxin biosynthesis gene, *OsYUC8*, which is induced by OsbZIP46 to promote auxin biosynthesis in compaction-stressed root, was reported to inhibit epidermal cell elongation and root elongation in rice [13,15]. However, the alfalfa homologs of *OsbZIP46* and *OsYUC8* were inconsistent with rice in responses to soil compaction (Figure S5). In fact, the overall auxin content remained unchanged upon compaction stress in the present study (Figure 7D). Both the contradiction of *YUC8* and the inconsistence of SAUR homolog expression in the present study indicate a complex signaling of auxin towards soil compaction in alfalfa. Moreover, that auxin acts as a key regulator of xylem development and a putative determinant of the vessel diameter is linked to the former discussed lignin biosynthesis pathway [46]. Taken together, we suggest that auxin may play a vital role in alfalfa responses to soil compaction, but its function mechanism must be investigated in more detail at the tissue and cellular levels.

PYR/PYL/RCAR, PP2C and SnRK2 are three core components of the ABA signaling pathway. The binding of ABA with PYR/PYL/RCAR leads to the block of the activity of PP2C, and then SnRK2s are activated and phosphorylate the downstream ABA-responsive transcription factors [58]. In the current research, several gene homologs of *PYL*, *PP2C* and *SnRK2* were identified as DEGs in CP-treated roots. Notably, the expressions of the three gene homologs were consistently down-, up- and down-regulated, respectively (Figure 7). In Arabidopsis, it was reported that the knockout of *AtPYR/AtPYL* results in strong ABA hyposensitivity during root growth and the transcriptional regulation of ABA-responsive genes [59], which is in line with the possible decreased ABA level. Moreover, PP2Cs and SnRK2s are, respectively, considered to be general negative and positive regulators of ABA signaling [58], which further evidences the weakened ABA signaling in the CP-treated alfalfa roots in the present study. However, the decreases in the ABA content and signaling in alfalfa are not in accordance with the rice scenario, which denotes ABA as a promising entry point to fish out other regulators and untangle the whole mystery in alfalfa.

Soil compaction lowers gas diffusion, causing ethylene to accumulate in root tissues and trigger hormone responses that restrict growth in Arabidopsis and rice [13,14]. This conclusion was supported by the moderate elevation in the ACC content in alfalfa (Figure 7F). the Arabidopsis AtFER-AtPIF3-AtPIEZO circuitry seems not to work in alfalfa, as we failed to identify any AtPIF3 homologs in the Zhongmu-4 genome, and no noticeable transcriptional changes were observed in the *AtFER* or *AtPIEZO* homologs. Moreover, we observed a significant increase in the JA content in the CP-stressed alfalfa roots. As far as we know, there is no report of JA function in plants’ acclimation to soil compaction, which may be taken into consideration in future research.

## 5. Conclusions

The growth of the perennial forage alfalfa is susceptible to soil compaction, but it is hard to obtain timely alleviation from tillage, so modification in germplasm is of great value. In this research, we systematically studied the effects of compaction intensity on alfalfa seedling growth and explored the capacity for genetically improving the plant performance. We found that a slight increase (about 1.08 times in the bulk density of the naturally loosened soil) in the soil bulk density is beneficial for alfalfa growth, but high-level compaction (more than 1.17 times in the bulk density of the naturally loosened soil) has a significant and comprehensive repression effect. The negative effect caused by intensive soil compaction is universal in different alfalfa germplasms, but there is still a distinct gap between them, indicating the requisite future research in breed improvement. The gene expression profiles implicated that the repression effect might mainly be derived from the reduction in phenylpropanoid and its derivatives, namely lignin and flavonoid biosynthesis, which resulted in modified cell development, especially in the cell wall and xylem vessel formation. Phytohormones like ABA, auxin and ethylene were supposed to play pivotal roles in the regulation of the overall responses. The markedly increased level of JA implies its role in further studies of alfalfa responses to soil compaction. We suggest that field management should avoid or reduce tillage methods that might cause the severe compaction of soil, and future research should be mainly devoted to functional validation and mechanism studies of candidate gene homologs in the key processes with the aim of breeding smarter germplasms.

## Data Availability

The raw sequence data reported in this paper have been deposited in the Genome Sequence Archive (Genomics, Proteomics & Bioinformatics 2021) in the National Genomics Data Center, the China National Center for Bioinformation/Beijing Institute of Genomics, the Chinese Academy of Sciences (GSA: CRA013571) and are publicly accessible at https://ngdc.cncb.ac.cn/gsa (accessed on 21 November 2023).

## Supplementary Materials

The following supporting information can be downloaded at https://www.mdpi.com/article/10.3390/plants13070953/s1, Figure S1: Phenotype and macronutrient analysis of alfalfa seedlings’ responses to soil compaction; Figure S2: Interpretation of transcriptome data by cluster, heatmap and volcano plot; Figure S3: qRT-PCR validation of transcriptome data; Figure S4: Contents of cytokinin, gibberellin, salicylic acid and jasmonic acid in alfalfa roots subjected to soil compaction; Figure S5: Expression levels of *YUC8* and *bZIP46* homologs in alfalfa roots subjected to soil compaction; Table S1: Physicochemical properties of soil used in this study; Table S2: Primers used in this study; Table S3: Quality information of RNA-Seq data; Table S4: Differentially expressed genes identified between control and soil-compaction-stressed alfalfa roots. Table S5: Significantly enriched Gene Ontology annotations of differentially expressed genes; Table S6: Cluster information of Gene Ontology biological process annotations.
