# Supplementary material for: Alfalfa Responses to Intensive Soil Compaction: Effects on Plant and Root Growth, Phytohormones and Internal Gene Expression
Source: Plants (Basel). 2024 Mar 26;13(7):953. doi: 10.3390/plants13070953 (PMC11013635; doi:10.3390/plants13070953)
Supplement: Supplementary file 1 [file plants-13-00953-s001.zip › Supplementary figure caption.pdf]

Figure S1: Phenotype and macronutrient analysis of alfalfa seedlings' responses to soil compaction. Phenotypical measurements: shoot fresh weight, root fresh weight, average root angle, root-to-shoot ratio. Macronutrient content: nitrogen (N), phosphate (P), potassium (K). DW, dry weight. Seedlings of different cultivars were grown in soil with bulk density of 1.2 g cm^{-3} (CK) and 1.5 g cm^{-3} (CP) for five weeks until imaging and measurement; $n = 3$ sets of at least 5 seedlings. Error bars represent the means ± SE. Asterisks represent significant differences between samples analyzed using Student's *t*-test: * $p < 0.05$; ** $p < 0.01$.

Figure S2: Interpretation of transcriptome data by cluster, heatmap and volcano plot. (A) Heatmap and sample cluster demonstration of differentially expressed genes. Euclidean distance of normalized read count was used in clustering. (B) Volcano plot of all genes detected in RNA-Seq study.

Figure S3: qRT-PCR validation of transcriptome data. TPM, transcripts per million; CK, 1.2 g cm^{-3} (bulk density); CP, 1.5 g cm^{-3}. Error bars represent means ± SE.

Figure S4: Contents of cytokinin, gibberellin, salicylic acid and jasmonic acid in alfalfa roots subjected to soil compaction. (A–F) Zeatin (ZT), trans-zeatin riboside (tZR), gibberellic acid (GA3), salicylic acid (SA), jasmonic acid (JA) and jasmonoyl-isoleucine (JA-Ile). CK, 1.2 g cm^{-3} (bulk density); CP, 1.5 g cm^{-3}. Error bars represent means ± SE. Asterisks represent significant differences between samples analyzed using Student's *t*-test: * $p < 0.05$.

Figure S5: Expression levels of *YUC8* and *bZIP46* homologs in alfalfa roots subjected to soil compaction. *YUC8L1–3* (*YUC8 LIKE 1–3*) and *bZIP46L1–3* (*bZIP46 LIKE 1–3*) are homologs of rice genes, *OsYUC8* and *OsbZIP46*, respectively. TPM, transcripts per million; CK, 1.2 g cm^{-3} (bulk density); CP, 1.5 g cm^{-3}. Error bars represent means ± SE. Asterisks represent significant differences between samples analyzed using Student's *t*-test: ** $p < 0.01$.
